# An *In Vitro* Study of Differentiation of Hematopoietic Cells to Endothelial Cells

**DOI:** 10.1155/2011/846096

**Published:** 2011-12-29

**Authors:** Qi Ru Wang, Bao He Wang, Wen Biao Zhu, Yan Hong Huang, Yi Li, Qi Yan

**Affiliations:** ^1^Department of Physiology, Xiangya Medical College, Central South University, Changsha 410078, China; ^2^College of Life Science, Hunan Normal University, Changsha 410081, China; ^3^Everett Medical Center, GHC, Everett, WA 98201, USA; ^4^Benaroya Research Institute and Departments of Biological Structure and Ophthalmology, University of Washington, Seattle, WA 98195, USA

## Abstract

Bone-marrow-derived endothelial progenitor cells (BM-EPCs) contribute to postnatal neovascularization and therefore are of great interest for cell therapies to treat ischemic diseases. However, their origin and characteristics are still in controversy. In this paper, we identified the origin/lineage of the BM-EPCs that were isolated from bone marrow mononuclear cells and differentiated with the induction of bone-marrow endothelial-cellconditioned medium (ECCM). BM-EPCs were characterized in terms of phenotype, lineage potential, and their functional properties. Endothelial cell colonies derived from BM-EPC were cultured with ECCM for 3 months. Cultured EPC colony cells expressed endothelial cell markers and formed the capillary-like network *in vitro*. EPC colony cells expressed differential proliferative capacity; some of the colonies exhibited a high proliferative potential (HPP) capacity up to 20 population doublings. More importantly, these HPP-EPCs expressed hematopoietic marker CD45, exhibited endocytic activities, and preserved some of the myeloid cell activity. In addition, the HPP-EPCs secrete various growth factors including VEGF and GM-CSF into the culture medium. The results demonstrate that these EPCs were primarily derived from hematopoietic origin of early precursor cells and maintained high proliferative potential capacity, a feature with a significant potential in the application of cell therapy in ischemic diseases.

## 1. Introduction

Bone marrow mononuclear cells (BMMNCs) contain endothelial progenitor cells (EPCs) valuable in cell therapy to enhance postischemic neovascularization. It has been shown that BMMNCs, which can be easily prepared from bone marrow extraction, have dramatic effects in the formation of neovascularization in ischemic diseases or tumor model [1–4]. In spite of extensive studies on EPC [3, 5–7], it is still quite challenging to induce all the potential EPCs from BMMNC or bone marrow into functional EPC due to the following reasons: (1) hierarchy of hematopoietic progenitors in bone marrow; (2) EPCs exist as a distinct cell type residing in the bone marrow stroma; (3) imperfect induction conditions *in vitro*. Extensive literature clearly documented the presence of EPCs in the bone marrow; it is important to elucidate the origin of the BM-EPC and continuously to improve strategies for isolating and characterizing the valuable EPC from BMMNC, thereby ultimately utilizing these EPCs for therapeutic purposes in ischemic and tumor diseases.

We have established and characterized a BMEC line in our lab [8]. BMEC-CM derived from the BMEC line culture enhanced the proliferation and differentiation of hematopoietic progenitors [9–11] and stimulated the proliferation of EPC [12, 13]. In this paper, we present the *in vitro* model for characterization of bone-marrow-derived EPC with the induction of BMEC-CM. Under this unique condition, a population of bone-marrow-derived EPC has been enriched and characterized. Our data demonstrate that these EPCs were primarily derived from hematopoietic origin of early precursor cells and maintained high proliferative potential (HPP) capacity, a feature with a significant potential in the application of cell therapy in ischemic diseases.

## 2. Methods

### 2.1. Culture of Endothelial Cell Colony

Murine bone marrow mononuclear cells (BMMNCs) were prepared. Bone marrow EC-cols were established using the previously described method with modification [12]. Briefly, one million BMMNCs were suspended in DMEM containing 20% FBS and 20% bone-marrow-endothelial-cell-conditioned medium (BMEC-CM, see below) and were seeded onto a fibronectin-coated well of a 24-well tissue plate. After 5 days in culture, cells were trypsinized and replated at low density of 2 × 10^4^ cells/well (6-well plate) or 10 cells/well (96-well plate). Each well of 96-well plate or 6-well plate contained 200 *μ*l or 2 mL DMEM with 20% FBS and 20% EC-CM, respectively. Cells were cultured at 37°C, 5% CO2 in a humidified incubator. Media were changed every week. Each well was examined for the growth of endothelial cells every 3 days. In most wells of 96-well plate, one colony was observed in each well. The wells that contained only one colony were selected for the study. In the well of 6-well plate, one big colony (>10,000 cells) was selected and the other endothelial cells and smaller EC-cols were removed with a policeman under microscope after 4 weeks in culture. As a result, only one EC-col was cultured in each well. The cell number of each colony was counted under microscope or counted with a hemacytometer.

### 2.2. Preparation of Bone-Marrow-Endothelial-Cell-Conditioned Medium

The murine bone-marrow-derived endothelial cell line was established in our laboratory [8]. One hundred thousand immortalized endothelial cells or fresh cultured EPC-derived cells were cultured in DMEM for 48 hours without serum. The conditioned medium from endothelial cell line was named BMEC-CM; the conditioned medium from EPC-derived cells was named EPC-CM. The conditioned media were collected and kept at −20°C before further use.

### 2.3. Immunofluorescence

Cells from colonies were immunostained with anti-murine CD31 IgG (Sigma), anti-von Willebrand factor (vWF) IgG (Dako), and anti-CD45 IgG (Biolegend), respectively, followed by secondary antibodies conjugated to FITC or Texas-red, using a standard procedure described previously. To assess lectin binding, biotinylated Ulex europaeus agglutinin-I (UEA-I) and streptavidin-Alexa Fluor 488 were applied. In addition, cells were incubated with 10 ug/mL DiI-Ac-LDL for the uptake of acetylated LDL assay as described previously [12].

### 2.4. Proliferative Potentials

Cell numbers of each colony cultured in the wells of 96-well plate were counted under microscope starting from day 7 of culture and followed up for 30 days. The number of cells per colony cultured in a well of 6-well plate was counted under microscope or with a hemacytometer every two weeks and followed up for 14 weeks. The data were analyzed for the description of the growth curve.

### 2.5. Vascular Network Formation

Fifty percent BMEC-CM plus 20% F-CM (fibroblast-cell-conditioned medium) was added into a well of 6-well plate in which a large colony has been cultured. Medium was replaced each week. Vascular network formation was monitored every day under an inverted microscopy.

### 2.6. Secreted Growth Factors

To analyze growth factors secreted into the culture media, cells derived from EC-cols, which have been cultured for more than one and half month in tissue culture incubator, were exposed to serum-free and growth-factor-free DMEM For 48 hours. Conditioned media were collected and analyzed for vascular endothelial growth factor and granulocyte-macrophage colony-stimulating factor (GM-CSF) by ELISA (R and D system). For evaluation of the biological activity of the secreted growth factors in the CM, the serum-free supernatant of EC colony cells (EPC-CM) was tested in the liquid culture system with variable concentrations of 0%, 5%, 10%, 20%, and 40%, respectively; the growth of endothelial cell colony was subsequently evaluated.

### 2.7. Endocytosis

Endocytosis assays were performed with EC-col-derived cells, macrophages, and HUVECs. The preparation of *Candida albicans* reagent was by the addition of *Candida albicans* to DMEM containing 20% FBS. Adherent cells derived from the EC colony (45d) in the 96-well plate were incubated with this solution for 2 hours in tissue culture incubator. Subsequently, cells were stained with Wright-Giemsa's staining. Numbers of *Candida albicans* internalized by each cell were counted under microscope. Macrophages and HUVECs were included as positive and negative controls, respectively, for endocytosis.

### 2.8. Statistical Analysis

Results were expressed as means ± SD. Levels of significance were determined using Student's *t*-test. *P* values less than 0.05 were considered statistically significant.

## 3. Results

### 3.1. Morphology and Phenotype of EC Colony

**Figure 1:**
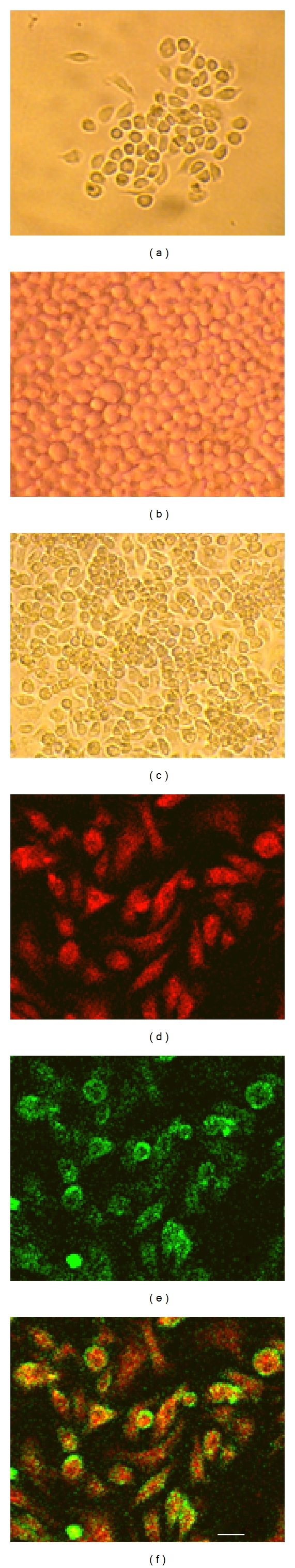
Bone-marrow-derived adherent cells were identified to be endothelial progenitor cells. After 5-day culture of BMMNCs with BMEC-CM, adherent cells were replated at low density. A representative of 10-day colony (a), 30-day colony (b), and 60-day colony (c) was shown, respectively. Round cells or spindle-shaped cells were observed in (a), (b), and (c). These cells were positive for vWF (d) and CD31(e). (f) is the merge of (d) and (e). Scale bar, 50 *μ*m.

BMMNCs were incubated in 20% BMEC-CM for 5 days, this CM-mediated induction significantly promotes the differentiation of the BMMNC towards EPC as shown in Figure 1. After the initial 5-day incubation, BMMNCs were replated in a low-density culture dish in the presence of BMEC-CM. EPC-derived colonies (EC-cols) were formed with variable sizes of colonies containing dozens to thousands of cells per colony.

**Figure 2:**
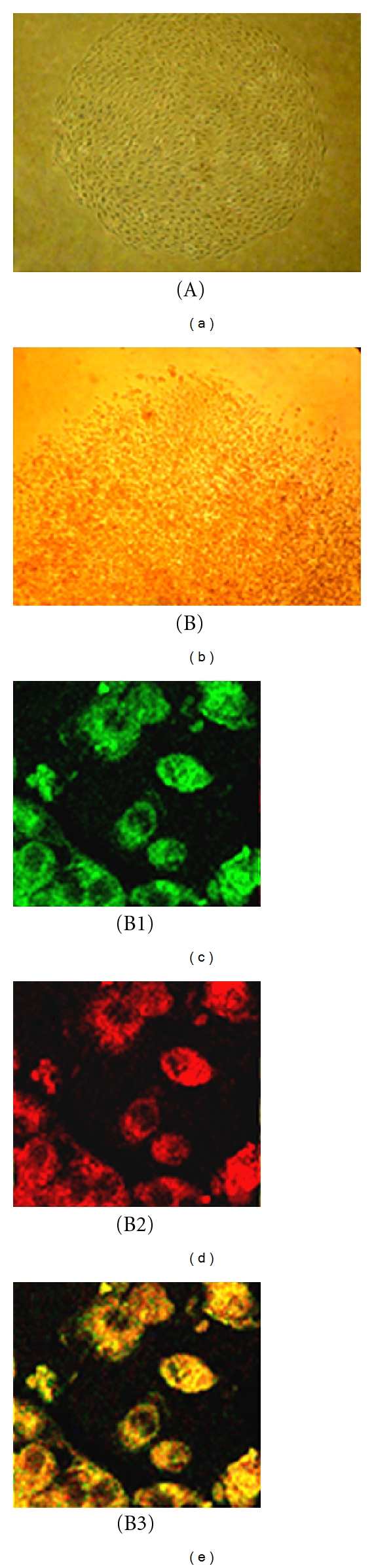
Bone-marrow-derived endothelial cell colonies. An eighteen-day colony (A) and 35-day colony (B) were shown. Cells from colony A were negative for CD45 and positive for EC markers (data not shown). Cells from colony B were positive for CD31(B1) and CD45(B2). (B3) is the merge of (B1) and (B2).

 An EPC colony, negative for CD45 and positive for EC markers, was identified in experiments of the primary EPC colony culture (Figure 2(A)). Interestingly, the CD45-EC+ colony was rarely found in the EPC colony culture with this *in vitro* condition. CD45-EPC colony can be derived from either angioblasts or mesenchymal stem cells (MSCs) in the bone marrow [14, 15]. We might speculate that this particular CD45-EC+ colony (Figure 2(A)) was derived from angioblast rather than MSC, because MSCs were completely inhibited by cytokines/growth factors presented in the BMEC-CM used for EPC colony culture; this has been demonstrated previously in our lab [12]. In contrast, most of the EPC colonies generated with this *in vitro* condition, regardless of cultures in the plate of 96 wells or 6 wells, were CD45+ EC+ (Figure 2(B)). Given the fact that EPCs derived from hematopoietic lineage express CD45+, it is thus logical to propose that the CD45+ bone marrow-derived EPCs are originated from hematopoietic lineage.

EPCs possess differential proliferative capacity depending on the hierarchy of hematopoietic progenitor that they are derived from. Quantitatively, we have observed that  671 ± 93.5  EPCs were derived from 1 × 10^6^ bone marrow mononuclear cells. For example, if the EPC is derived from a myeloid progenitor or monocyte, this EPC has limited proliferative capacity, resulting in a small colony [16]. On the contrary, if the EPC is derived from hematopoietic stem cell or an undifferentiated early precursor in this lineage, this EPC has potent proliferative capacity, resulting in a large colony. The percentage of high proliferative potential EPC colonies is 1-2% among all the EPC colonies. This discrepancy of EPC proliferative capacity has also been attributed to the hierarchy of hematopoietic progenitors in bone marrow [17]. Next, we designed experiments to evaluate the EPC proliferation capacity. 

### 3.2. EPC Proliferation

**Figure 3:**
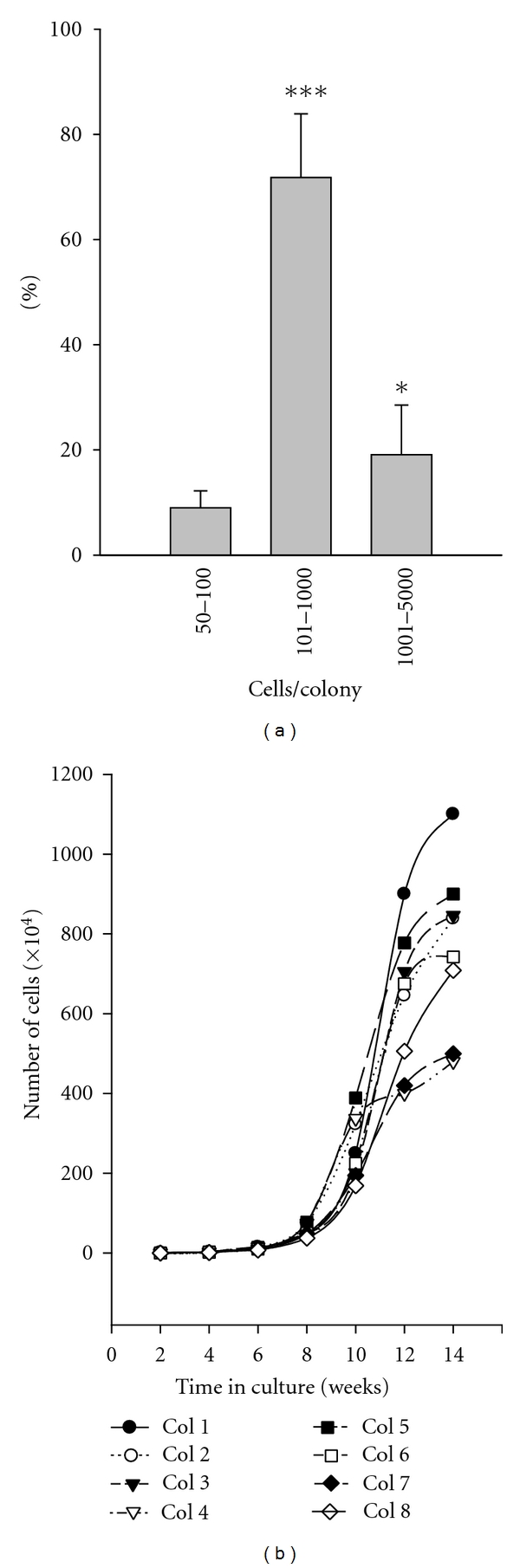
Proliferative capacity assay of bone-marrow-derived EPC. (a) Distribution of different sizes of EC-col after 21 days of culture. Each bar represents the mean ± SD of 3 experiments. *P < 0.05, ***P < 0.001 in comparison to the group of 50–100 cells. (b) Growth curve of eight large EC-cols. The number of cells from each colony was counted every 2 weeks for 14 weeks.

Proliferative capacity of a single endothelial progenitor cell was evaluated by the colony size. After 5-day culture of BMMNC with BMEC-CM, cells were tripsinized and replated for evaluation of their proliferative capacity. To examine colony size of 30-day culture, cells were plated at 10 cells/well of 96-well plates. At this low cell-plating density, one colony per well was achieved in most of the wells of 96-well plates. To examine the high proliferative potential colony, 2 × 10^4^ cells/well of 6-well plate were plated. After 4 weeks in culture, one large endothelial cell colony was selected and allowed to grow in one well (see Section 2). Eighty-two single cell colonies were formed from wells of 96-well plate, and 8 high proliferative potential colonies were observed from 6-well plates. Among the 82 colonies, 72% of single EPC gave rise to colonies containing cells ranging from 101 to 1000 cells, 9% ranging from 50–100 cells, and 19% ranging from 1001 to 5000 cells or more in a period of 3 weeks of culture (Figure 3(a)). The cell number/per colony was (740.97 ± 230.70) × 10^4^ cells in the eight high proliferative potential colonies in a period of 14 weeks of culture (Figure 3(b)). Samples of these colony-derived cells were reexamined for cell markers by immunofluorescence, they are positive for EC markers and positive for CD45. These EPCs were expanded for 20 population doublings.

### 3.3. Angiogenic Capacity of the Colony Cells

**Figure 4:**
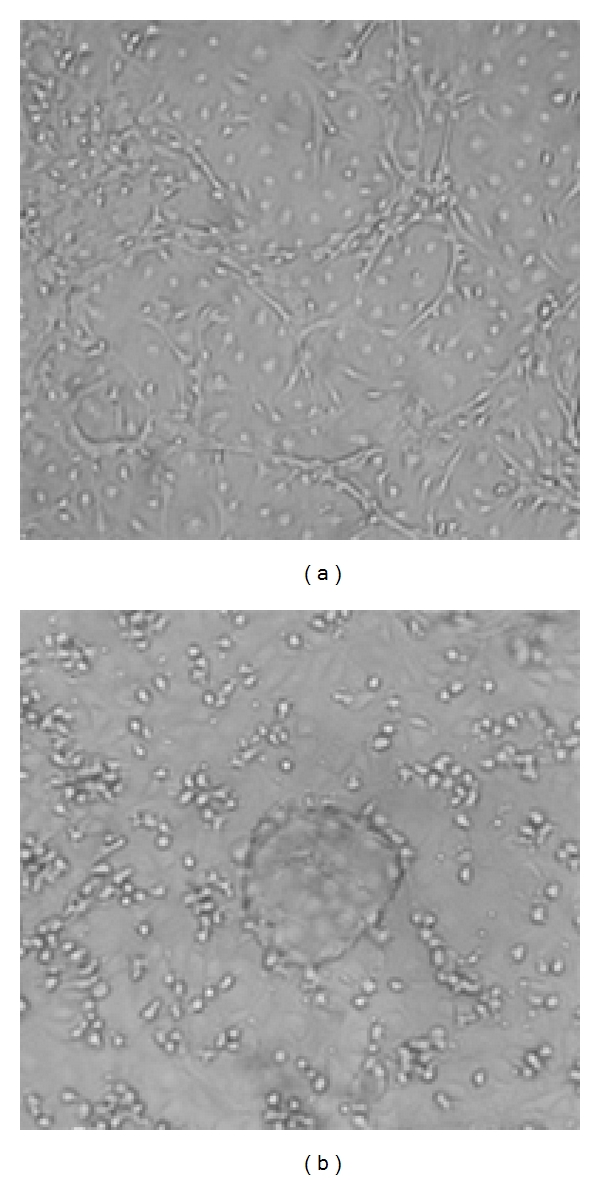
Cells of EPC colony (CD45+ EC+) derived from BMMNCs (a) formed capillary-like network when exposed to 50% EC-CM and 20% F-CM for 2 days and (b) when exposed to VEGF for 2 days.

EPC colony cells derived from BMMNC showed capacity of forming capillary-like network structure *in vitro*. We have found that 25–37% of EPC colonies have the capacity to form capillary-like structure with appropriate culture condition. EPC colony cells (45d) formed capillary-like structure when exposed to 50% BMEC-CM plus 20% F-CM for 2 days (Figure 4(a)).  15 ± 2  capillary-like structures per 20 × microscopic field were formed. In contrast, the capillary-like structure did not form (0 to 1 capillary-like structure per field) when exposed to VEGF matrigel condition for the same time points.

### 3.4. Growth Factors Produced by EPC In Vitro

**Figure 5:**
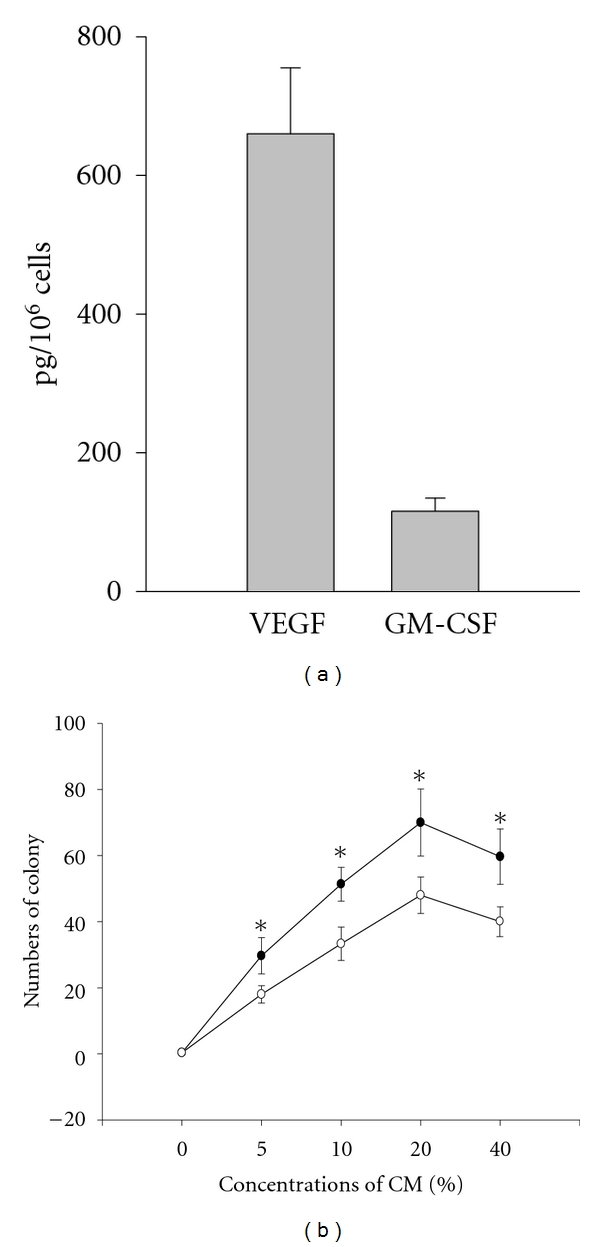
Analysis of cytokines in EPC-CM. The culture media of EPC colonies were collected after 48-hour incubation in serum-free condition. (a) Concentrations of cytokines in supernatant were measured by ELISA. (b) Ten thousand cells were cultured in wells of 24-well plate with EPC-CM (closed circle) or HUVEC-CM (open circle) of the following concentrations: 0%, 5%, 10%, 20%, and 40%, respectively. The number of endothelial cell colonies was counted after 14-day culture. The mean ±SD of triplicates-presented at each point in comparison to HUVEC-CM group. *P < 0.05. VEGF: vascular endothelial growth factor, GM-CSF: granulocyte-macrophage colony-stimulating factor.

BMMNC-derived EPCs were cultured in serum-free and BMEC-CM-free medium. The cultured medium was harvested after 48-hour incubation. We evaluated the level of cytokines secreted by EPC using ELISA assay. Over a 48-hour culture, EPCs secreted VEGF of 660 ± 95.39 pg/10^6^ cells/mL and GM-CSF of 115.66 ± 18.87 pg/10^6^ cells/mL (Figure 5(a)). The effect of various concentrations of the supernatant (EPC-CM) on EC-col formation was further analyzed. The EC-cols were formed in the presence of EPC-CM. There was a dose-dependent effect of EPC-CM on the growth of EC-cols (Figure 5(b)). In comparison to HUVEC-CM, EPC-CM showed a stronger stimulation for the formation of EC-cols (Figure 5(b)).

### 3.5. Endocytic Capability

**Figure 6:**
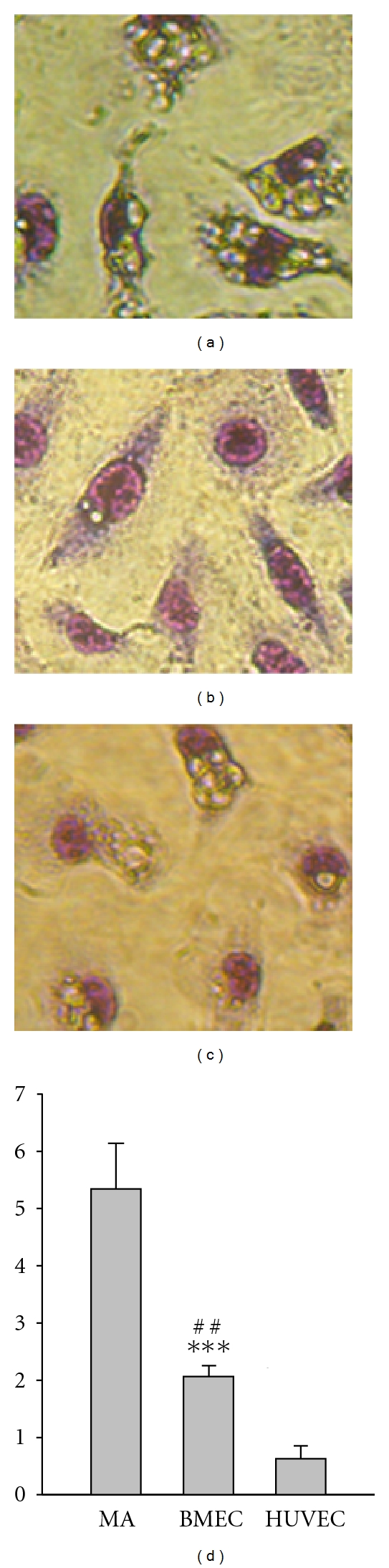
Endocytosis assay. The number of *Candida albican* internalized by each cell was counted under microscope. (a) Macrophages (b) Cells from BMMNC-derived EPC-cols. (c) HUVEC. (d) The number of internalized *Candida albicans* per cell was shown for each cell type. Bars represent mean ± SD of the number of *candida albicanin* in each type of the cells of three experiments. MA: macrophages. BMEC: bone marrow endothelial cells from EPC-colony (45d). HUVEC: human umbilical vein endothelial cells. ^##^ *P* < 0.01 (BMEC versus MA), ***P < 0.001 (BMEC versus HUVEC).

A critical feature of monocytes is the endocytic activity. EPCs derived from CFU-GM or myeloid lineage maintain strong endocytic activity. We examined the endocytic capability of EPC-col-derived cells. EPC-col-derived cells were incubated with *Candida albicans*, and the number of the *Candida albicans* internalized by each cell was counted. Macrophages and HUVEC were used as controls. The result showed that cells derived from EPC-cols internalized significantly more *Candida albicans* than HUVEC (*P* < 0.001), but less than macrophages (*P* < 0.01). Monocytes/macrophages are derived from myeloid lineage and CD45+, exhibiting a strong phagocytic activity (Figure 6(a)). Conversely, HUVECs are derived from angioblasts [14] and CD45− [18], exhibiting a weak phagocytic activity (Figure 6(c)). In fact, these EPCs maintained some of the phagocytic feature, one of the critical characteristics of hematopoietic lineage cells. More importantly, this phagocytic activity demonstrated by these EPCs argues against the possibility of their angioblast origin.

## 4. Discussion

In this study, with the induction of BMEC-CM, BMMNC gave rise to cell colonies that expressed EC markers, incorporated Ac-LDL, reacted with endothelial specific Ulex europaeus lectin; showed endothelial morphology, exhibited a high proliferative capacity, and formed capillary-like structures, all of these indicating they are EPC.

**Figure 7:**
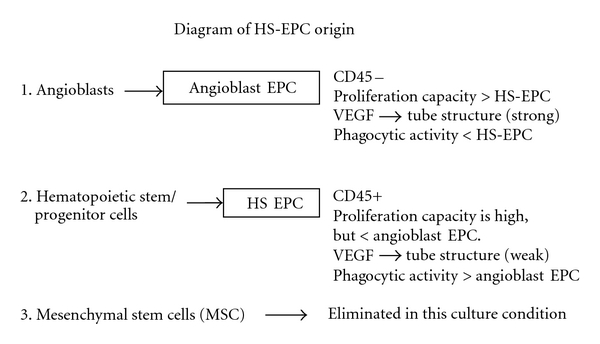
A diagram showing a differentiation pathway for HS-EPC derived from bone marrow origin. The hematopoietic stem/progenitor cells were selectively adhered to the culture plates and proliferated/differentiated in the presence of BMEC-CM *in vitro*. EPCs generated in this *in vitro* model most likely are originated from hematopoietic stem cells or their early progenitors rather than from angioblsts or mesenchymal stem cells. HS-EPC: hematopoietic stem cell or early-hematopoietic-progenitor-cell-derived EPC.

A variety of stem cells/progenitor cells in bone marrow can be induced and differentiated into EPC *in vivo* and *in vitro*. The rare hemangioblasts and their progeny angioblasts [14, 19–22], the hematopoietic stem cells [23] and their progeny of myeloid lineage [24–26], and the multipotent adult progenitor cells and/or MSC [15] are the primary sources to give rise to EPC. In this study, the growth of MSC from BMMNC was completely inhibited as described in our previous report [12]. Therefore, the EPC generated in this report (hereafter termed HS-EPC) could be originated either from hemangioblasts, angioblasts, and their progeny, or from hematopoietic stem cells and their progeny (Figure 7). It is highly unlikely that HS-EPCs are originated from angioblasts for the following reasons: (1) the EPCs derived from angioblasts have higher proliferative capacity relative to HS-EPC. For example, the cord-blood-derived HPP-EPC could be expanded for at least 100 population doublings and yield 10^10^ to 10^12^ cells per EPC after 90-day incubation [27]. HUVECs and HAECs derived from angioblasts can be passaged for at least 40 population doublings *in vitro* [18]. In contrast, HS-EPC can be expanded for 20 population doublings and yield 7 × 10^6^ cells per EPC after 14-week incubation (Figure 3(b)). (2) EPC derived from angioblasts responded well to VEGF; VEGF is a potent stimulator for the formation of capillary-like structure for EPC derived from angioblasts [27]. On the contrary, HS-EPC responded poorly to VEGF in the formation of capillary-like structure (Figure 4). Moreover, EC-CMs or F-CMs rather than VEGF were more potent stimulators for the formation of capillary-like structures on HS-EPC cells (Figure 4). (3) Cells derived from angioblasts showed a weak phagocytic capability (Figure 6, HUVEC), but HS-EPC exhibited a significantly higher phagocytic ability than HUVEC (angioblast progeny. (4) EPCs derived from angioblasts or MSC were negative for CD45, whereas HS-EPCs were positive for CD45 (Figure 2(b)). It is most likely that HS-EPCs are originated from hematopoietic stem cells and their early progeny (Figure 7). It has been reported that the hematopoietic stem cells and their progeny of myeloid lineage have been induced to differentiate into endothelial cells [2, 16, 23, 24, 28]. Pleiotrophin might induce transdifferentiation of monocytes into functional endothelial cells [29]; likewise, TNF*α* also significantly facilitates the endothelial differentiation of myeloid cells *in vitro* and *in vivo* [30]. Madlambayan et al. [31] demonstrate that myeloid progenitor cells directly participate in new blood vessel formation in response to SDF-1*α*. They report that secondary transplantation of single hematopoietic stem cells showed that HSCs are a long-term source for neovasculogenesis. Our experiments provide evidence that hematopoietic cells can differentiate into endothelium in a liquid culture with EC-CM. We believe that EC-CM contains various factors including VEGF, SDF-1, and TNF-*α* and unknown factors that play a synergism effect in the induction of hematopoietic cells to endothelial cells. 

A common feature of EPC derived from myeloid linage and monocytes is their limited proliferative capacity [16, 32]. Interestingly, HS-EPC, positive for CD45, exhibited much higher capacity for proliferation in comparison to EPC derived from myeloid linage cells; for example, one HS-EPC yields about 7 × 10^6^ EC (Figure 3(b)), only hematopoietic-stem-cell-/early-progenitor-originated and differentiated endothelia lineage cells can possess such high proliferative capacity. This high proliferative capacity is indeed the characteristic of stem/precursor cell of hematopoietic lineage. We believe that the various cytokines and unidentified molecules secreted by the BMEC line (used for the preparation of BMEC-CM) [9] and their synergistic effects played a significant role in promoting the adherence of hematopoietic stem cells in the initial BMMNC culture; moreover, these factors not only facilitate the survivor, proliferation, and differentiation of stem cell/progenitor, but also induce stem cells to differentiate towards EPC lineage and stimulate their proliferation [12, 13]. The secreted factors/molecules preserved the characteristics of hematopoietic stem cells during the differentiation towards EPC, such as high capacity of proliferation. Thus, one hematopoietic stem cell or progenitor cell can result in million of endothelial cells under the induction of BMEC-EM. This phenotype is extremely important given their potential in repair in ischemic diseases. For example, a recent study showed that after intracoronary transplantation of bone marrow cells enriched in CD34/CD45+ and CD133/CD45+, patients with ischemic heart disease showed a significantly reduced size of infract area and an increased global ejection fraction as well as infract wall movement velocity [33]. Our study provides increasingly important insight for the treatment of ischemic disease by using HS-EPC. This method for isolating EPC is quite unique because it induced the early hematopoietic stem cells rather than late differentiated progenitors; thus, the high proliferative potential capacity is a trademark of this *in vitro* isolation technique, a critical feature in translational research in ischemic diseases. To further categorize the specific cell markers expressed on the HS-EPC is important for the sorting of this unique population of EPC. 

In this paper, we present an *in vitro* model of cell differentiation from hematopoietic stem/progenitor cells in BMMNC to endothelial linage. Given the advantage of easy access of bone marrow tissue and negative immune rejection by using self-bone-marrow cells, HS-EPC would be an important source for cell therapy in ischemic diseases. It is thus important to further elucidate the mechanisms of hematopoietic stem cell differentiation to endothelial linage.
